# Peristomal Skin Complications Are Common, Expensive, and Difficult to Manage: A Population Based Cost Modeling Study

**DOI:** 10.1371/journal.pone.0037813

**Published:** 2012-05-24

**Authors:** Søren Meisner, Paul-Antoine Lehur, Brendan Moran, Lina Martins, Gregor Borut Ernst Jemec

**Affiliations:** 1 Stoma Care Clinic, Bispebjerg University Hospital, Copenhagen, Denmark; 2 Institute of Digestive Diseases, University Hospital of Nantes, Nantes, France; 3 Pelican Cancer Foundation, North Hampshire Hospital, Basingstoke, United Kingdom; 4 London Health Sciences Centre, London, Ontario, Canada; 5 Department of Dermatology, Health Sciences Faculty, Roskilde Hospital, University of Copenhagen, Copenhagen, Denmark; The University of Queensland, Australia

## Abstract

**Background:**

Peristomal skin complications (PSCs) are the most common post-operative complications following creation of a stoma. Living with a stoma is a challenge, not only for the patient and their carers, but also for society as a whole. Due to methodological problems of PSC assessment, the associated health-economic burden of medium to longterm complications has been poorly described.

**Aim:**

The aim of the present study was to create a model to estimate treatment costs of PSCs using the standardized assessment Ostomy Skin Tool as a reference. The resultant model was applied to a real-life global data set of stoma patients (n = 3017) to determine the prevalence and financial burden of PSCs.

**Methods:**

Eleven experienced stoma care nurses were interviewed to get a global understanding of a treatment algorithm that formed the basis of the cost analysis. The estimated costs were based on a seven week treatment period. PSC costs were estimated for five underlying diagnostic categories and three levels of severity. The estimated treatment costs of severe cases of PSCs were increased 2–5 fold for the different diagnostic categories of PSCs compared with mild cases. French unit costs were applied to the global data set.

**Results:**

The estimated total average cost for a seven week treatment period (including appliances and accessories) was 263€ for those with PSCs (n = 1742) compared to 215€ for those without PSCs (n = 1172). A co-variance analysis showed that leakage level had a significant impact on PSC cost from ‘rarely/never’ to ‘always/often’ p<0.00001 and from ‘rarely/never’ to ‘sometimes’ p = 0.0115.

**Conclusion:**

PSCs are common and troublesome and the consequences are substantial, both for the patient and from a health economic viewpoint. PSCs should be diagnosed and treated at an early stage to prevent long term, debilitating and expensive complications.

## Introduction

Creation of a stoma is a commonly performed and crucial component of abdominal and general surgical practice. The consequences for the patient can however be both complex and life threatening [1]. Stoma complications occur with a high frequency in spite of careful pre-operative planning, continuously improving surgical technique and extensive surgical experience [2]. It has been suggested that the complication rate appears to have in fact remained the same for the last 50 years [3], despite major advances in other aspects of medical and surgical care over the same period. Stoma complications include necrosis, leakage, granuloma formation, retraction, stenosis, prolapsed and parastomal hernia, and peristomal skin diseases [4]. While considerable attention has been focused on the surgical complications in the published literature, the consequences of peristomal skin complications (PSCs) have attracted less notice. PSCs are a constant challenge for a great majority of individuals with a stoma. It is the most common post-operative complication following creation of a stoma. Various studies have reported a PSC rate ranging from 18–60% [5]–[8] and a recent study reported that peristomal skin problems account for about 40% of all visits to stoma care nurses [9], suggesting that these problems play a greater role in the life of people with stomas than generally acknowledged [10]. The risk is life long, but the incidence of complications is highest in the first five years after operation [11] where a considerable reduction in stoma diameter and height is generally the norm – hence requiring adjustment of the appliance to minimize the risk of leakage and PSCs.

There are several challenges in the assessment of PSCs, one being to define the exact prevalence. The wide variation in reporting the complications may be due to the less than systematic assessment of the peristomal skin by different groups of health care professionals who seldom communicate on aspects of PSCs. In 2008 Martins et al developed the Ostomy Skin Tool (OST) using a simple and standardized approach for assessing peristomal skin [12]. The intention was to improve patient monitoring and optimize communication between health care professionals. Currently, the OST is a widely used and accepted tool translated into several languages. In Japan, this instrument has been implemented as a national standard tool. Recently the tool was validated and shown to be reliable [13], [14].

Another challenge is to objectively assess the impact of PSCs. One method of doing this could be to assess cost, either in terms of utilities such as time [15] or actual monetary cost. Therefore the aim of the present study was to create a model for cost assessment of PSCs using the OST as a reference. These costs are important to know for surgeons creating a stoma and for other stoma care professionals as the quality of stoma creation, function, and stoma care has a life-long direct impact on costs for the patients and health care systems at a level never previously defined in a similar approach. Finally the model was applied on a large real-life data set from people with ostomies to assess the prevalence and financial burden of PSCs.

## Materials and Methods

Initially a literature search was performed to find published relevant costs, cost estimations or health economic models on PSCs, however no relevant data were identified. Hereafter it was decided to create a model for cost estimations of PSCs.

### Model design for cost estimation of PSCs

**Figure 1:**
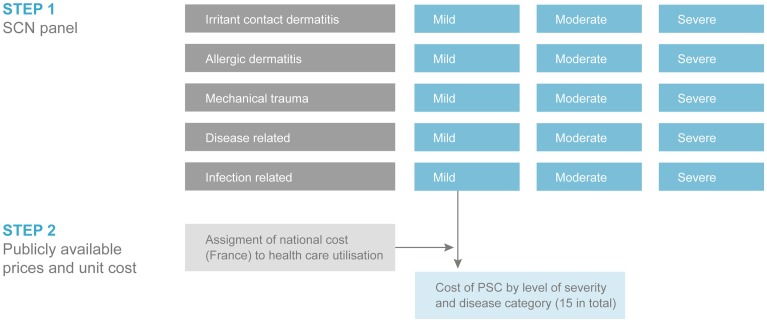
Model design for cost estimation of PSC.

The structure of the model (Figure 1) was based on contributions from three stoma expert panels using the validated OST as reference for determination of diagnosis categorization, severity and care of PSCs. A seven week treatment period was determined based on previous results from a recent study suggesting that clinically significant improvement of PSCs can be expected during a 6–8 week treatment period [7].

### The Ostomy Skin Tool (OST)

The OST is a standardized assessment tool developed with the aim to help health care professionals in evaluating and monitoring the condition of peristomal skin reliably and accurately [12]–[14]. The OST generates an objective score based on clinical observation of three domains: discoloration (D), erosion/ulceration (E), and tissue overgrowth (T). The three domains are scored according to the extent of the involved peristomal area and the severity of change in the skin. The combined score, or DET score, is in the range from 0–15 where 0 represents normal skin and 15 the worst combination of severity and extent. The OST also contains a full description of clinical signs for five diagnostic categories of PSCs and a care guide for each of the categories..Contact dermatitis and allergic dermatitis are two subcategories of an overall ‘chemical irritation’ category and the last three diagnostic categories are: mechanical trauma, disease related, and infection related. A simplified use of the DET score has been suggested [14] introducing three levels of severity ‘mild’ (DET<4), ‘moderate’ (DET≥4<7), and ‘severe’ (DET≥7).

### Consensus of appropriate PSC treatment (Step 1)

Best local practice varies from country-to-country and site-to-site. To develop an appropriate global understanding of a treatment algorithm to be used in a cost analysis, 11 highly skilled stoma care nurses from 9 countries were identified for interviews based on their daily practice with PSCs and their experience with the OST. The stoma care nurses were informed on the purpose of the study by e-mail and acceptance to participate were confirmed by e-mail. The nurses were interviewed face-to-face expressly for this study and informed consents were re-obtained verbally ahead of the interviews. The verbal consent was transcribed to interview notes. Before the interviews the stoma care nurses were introduced and trained in the simplified DET scale of 3 severity categories ‘mild’, ‘moderate’, and ‘severe’ which together with the OST care guide were used as a common framework in the interviews. The collection of interviews and analysis hereof were handled anonymously.

The stoma care nurses' recommendations for appropriate treatment for each case of ‘irritant contact dermatitis’, ‘allergic dermatitis’, ’mechanical trauma’, ‘disease related’ or ‘infection related’ for all three levels of severity (mild, moderate and severe) were recorded (see example Table 1). Health care spending was captured for health care visits, changes in type or use of appliances, medication, and surgery. The same interview questions were given to a dermatologist with special expertise in stoma care and the compilation of all interviews formed a global understanding of average treatment for every diagnostic category and severity level of PSC (15 subcategories in total). Complete treatment algorithms are available in Table S1.

**Table 1:** Example of recommended management of ‘moderate’ irritant contact dermatitis.*

Moderate irritant contact dermatitis	
Freq. (%)	Health Care Intervention
100	SCN consultation
51	2^nd^ SCN consultation
15	3^rd^ SCN consultation
6	Topical corticosteroid therapy

* Based on interviews of expert stoma care nurse (SCN).

### Assignment of cost for health care spending (step 2)

France was chosen as the country of reference because the cost of appliances is independent of the manufacturer. A unit cost was assigned for each treatment item to determine the cost induced by each subcategory of PSC. In France e.g., a typical treatment with topical corticosteroid costs 2.57€ and a stoma care nurse visit is estimated to cost 15€. The French unit costs for all products applied in this model can be found in Table S2.

### The DialogueStudy

To determine the distribution of treatment costs for the different diagnostic categories of PSCs and level of severity, the model was applied on data from the DialogueStudy (DS). The DS is the largest study ever undertaken in stoma care practice with more than 3000 people with a stoma enrolled from 18 countries [16]. The results from the DS provided a wealth of data on PSCs combined with leakage, ostomy appliance performance and quality of life assessed with the validated Stoma-Quality of Life (QoL) questionnaire [17]. In the DS, the participants' PSCs were assessed using the OST. Baseline data of gender distribution, type of stoma (colo-, ileo- or urostomy), mean age and mean time since surgery and presence of PSCs are listed in Table 2 and Table 3. In the model patients with multiple causes of PSCs or cause recorded as ‘other’ were assigned an imputed cost equal to the weighted average cost for a patient with known causes of PSCs with the same level of severity.

**Table 2:** Patient characteristics DS.

		Participants (N)	%
**Gender**	Males	1474	49
	Females	1541	51
**Type of stoma**	Colostomy	2015	67
	Ileostomy	954	31
	Urostomy*	46	2
**PSC**	Yes	1742	60
	No	1175	40

* People with urostomies were not enrolled in all countries. DS = Dialogue Study. PSC = Peristomal Skin Complication.

**Table 3:** Patient characteristics DS.

	Mean ± SD
**Mean age (years)**	63.2±14.3
**Time since surgery (years)**	5.9±7.9

### Sensitivity analysis (Tornado diagram)

**Figure 2:**
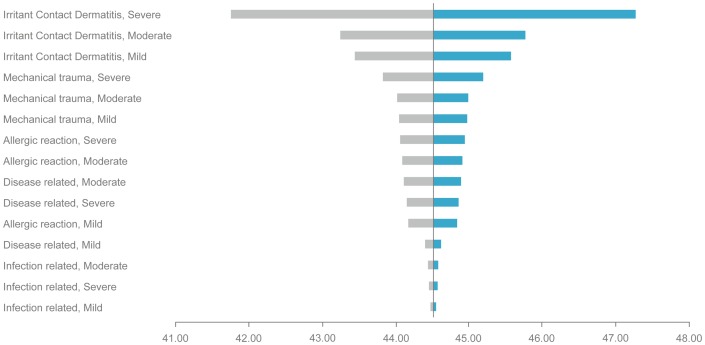
Sensitivity analysis of ±20% change in PSC cost presented in Tornado diagram.

Treatment cost estimation of PSCs was based on typical treatment patterns and did not rely on observed health care utilization. In order to test how robust the PSC cost estimates were with respect to the unit cost established by this method, a sensitivity analysis was conducted. Each of the 15 categories listed in Table 4 were varied by ±20% and the PSC cost estimates were re-established for baseline population in the DS, freezing the cost for the 14 other categories. The resulting 2×15 estimates are depicted in a Tornado diagram Figure 2. The Tornado diagram illustrates which of the 15 estimated category cost has the largest impact on the overall PSC cost estimate.

**Table 4:** Cost of managing a case of PSC by cause and severity.*

Diagnostic category	Mild	Moderate	Severe
Irritant contact dermatitis	20.86	24.89	152.19
Allergic reaction	46.92	68.02	106.23
Mechanical trauma	18.63	23.30	113.93
Disease related	40.45	87.91	195.82
Infection	35.39	49.24	167.69

* Mild: DET score<4; Moderate: DET Score<7; Severe: DET score≥7. DET = Discoloration, Erosion/Ulceration, Tissue-overgrowth. PSC = Peristomal Skin Complication. Cost estimations are based on French unit cost in 2011 (Euro).

### Statistical methods

The baseline PSC treatment costs are analyzed using a linear normal based analysis of covariance (multivariate analysis). PSC treatment costs are furthermore analyzed using a logarithmic transformation. Transforming estimates back, relative differences are obtained instead of absolute differences.

The covariates considered were:

- Type of ostomy
- One or two piece appliance
- Convex or non-convex baseplate
- Frequency of clinic visit to SCN or doctor
- The baseline leakage level (on a 3-level scale)
- Time span since stoma creation
- Reason for the stoma creation
- Permanency of stoma
- Age and gender of the patient
- Country and center treating the patient

The same sets of covariates were used for total and PSC cost analysis.

### Ethic statement

No ethic approval was obtained for the study as the study design is based on treatment algorithms, cost estimations and analysis of already published data from the Dialogue Study.

The stoma care nurses were informed on the purpose of the study by e-mail and acceptance to participate were confirmed by e-mail. The nurses were interviewed face-to-face expressly for this study and informed consents were re-obtained verbally ahead of the interviews. The verbal consent was transcribed to interview notes.

## Results

The model created was used to estimate the treatment costs for managing an average case of PSC for all levels of severity and diagnostic categories of PSC for a 7 week treatment period. The results are listed in Table 4 and are presented in euros based on French unit costs in 2011.

The treatment cost of severe cases of PSC increased 2–5 fold for the different categories of PSCs compared with mild cases of PSCs. The change in cost from mild to moderate cases showed an additional cost ranging from 4–47€ with ‘disease related’ PSCs at the highest end.

**Figure 3:**
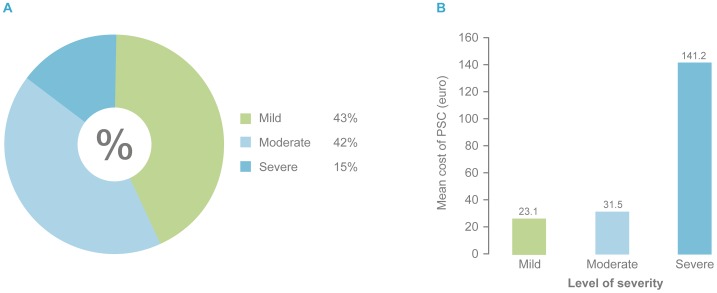
Cost of PSC according to level of severity (DS). PSC: Peristomal Skin Complication. DS: Dialogue Study.

**Figure 4:**
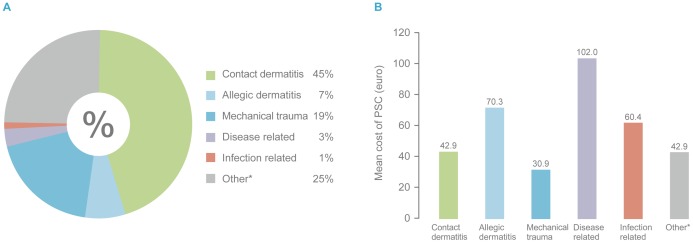
Cost of PSC according to diagnostic categories (DS). *Other: Was assigned an imputed cost equal to the weighted average cost for a patient with known cause of PSC with the same level of severity. PSC: Peristomal Skin Complication. DS: Dialogue Study.

The model was applied on the real-life baseline data from the DS. The treatment cost of a PSC was calculated for each patient where treatment of a patient with normal skin was assigned a treatment cost of zero. Based on the assumption that the numbers of appliance (baseplate and pouches) changes were the same for patients with and without PSCs, the total average cost for a 7 week treatment period (including appliances and accessories) was 263€ (n = 1742) for those with PSCs and 215€ (n = 1172) for those without PSC. Forty-three percent suffered from a ‘mild’ PSC, 42% had a ‘moderate’ PSC and 15% percent suffered from a severe PSC (Figure 3A). The treatment cost for the severe group was 6.1 fold higher compared with ‘mild’ cases and 4.5 fold higher compared with moderate cases (Figure 3B). The distribution of underlying causes of PSCs are shown in Figure 4A and the estimated treatment cost in Figure 4B. ‘Allergic dermatitis’, ‘disease related’ and ‘infection related’ had the highest estimated treatment cost (70.3€, 102.0€ and 60.4€ respectively).

In the DS, 58% of the participants had their stoma created as part of their treatment for cancer. The treatment cost of PSCs were in the same range [24–35.4€] independent of the underlying reason for stoma creation. DS participants with PSCs had an ileostomy or colostomy in 35% and 65% of the cases respectively. The average treatment cost for a patient with an ileostomy was 32.1€ and 24.0€ for a patient with a colostomy (Table 5).

**Table 5:** Cost of PSC (euro).

		N	%	Treatment cost mean (€)	Total treatment cost^1^ mean (€)
**Type of stoma**	Colostomy	1125	65	24.0	244.2
	Ileostomy	617	35	32.1	241.6
**Reason for stoma**	Cancer	949	55	24.2	242.4
	Ulcerative colitis	211	12	26.9	236.2
	Crohns disease	170	10	34.1	246.3
	Diverticulitis	123	7	35.4	254.8
	Other	268	16	27.8	246.5

1 Including appliances and accessories. PSC = Peristomal Skin Complication.

**Figure 5:**
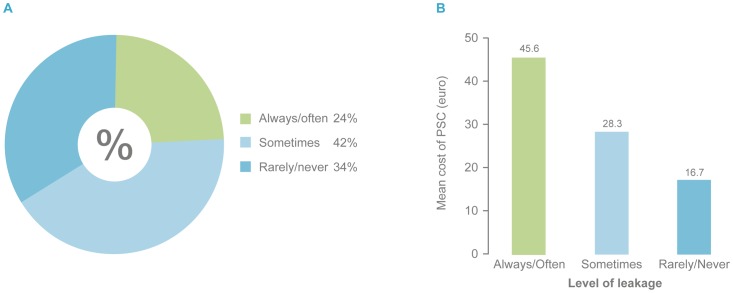
Cost of PSC according to leakage level (DS). PSC: Peristomal Skin Complication. DS: Dialogue Study.

Twenty-four percent of the participants in the DS experienced ‘always/often leakage’ and had the highest estimated cost (45.6€) compared with the group who ‘sometimes’ experienced leakage (28.3€) or ‘rarely/never’ (16.7€) (Figure 5A and B).

A co-variance analysis was performed to test for factors influencing the treatment cost of PSCs at baseline. Of the different variables tested leakage had a high statistically significant impact on the treatment cost of PSCs. From ‘rarely/never’ to ‘always/often’ there was a statistically significant impact on PSC treatment cost (p<0.00001) with a difference in an average cost of 29€ and from ‘sometimes’ to ‘rarely/never’, p = 0.0115 with an estimated difference in average cost of 11€. Furthermore, a borderline significant impact (p = 0.0422) was seen for ‘year of age at baseline’.

A sensitivity analysis of ±20% change in treatment cost for the 15 categories of PSCs was performed to assess the robustness of the model and the estimated cost related to PSCs. The outcome of the analysis is presented as a Tornado diagram in Figure 2. The results show that changes in the cost of a severe irritant contact dermatitis has the largest impact on the cost of PSCs. However the PSC cost estimate is robust to changes in the 15 subcategories; for instance the PSC cost changed 0.3% for a 1% change in the cost of a severe irritant contact dermatitis (at the top of the Tornado diagram), and less with any of the other 14 subcategories.

## Discussion

Surgical formation of a stoma is a significant clinical procedure in numerous ways, with stoma patients facing emotional, physical and social challenges. Stoma related complications occur frequently creating specific care problems and may have a major impact on the outcome in terms of patients' coping with their new life style. Living with a stoma is a challenge not only for the patient and their relatives and carers but also for society as a whole. In the long term it may be associated with increased absence from work due to illness, or to the need for early retirement, both significant health-economic burdens. The most common post-operative complication is PSCs [4]. The degree of peristomal skin irritation may range from that of a mild peristomal dermatitis to full-thickness skin necrosis and ulceration. A mild skin disorder if not taken seriously can rapidly progress into a more severe condition requiring medical action [6]. It is imperative that people with a stoma regularly check the peristomal skin and seek professional advice in a timely manner if deterioration in skin condition is observed.

In the current climate of limited available resources it is important to be aware of the most optimal use of resources. The aim of the present study was to create a model for cost estimation of PSCs. Interviews formed the basis for a model treatment algorithm for ‘mild’, ‘moderate’, and ‘severe’ cases of PSCs in five different underlying disease categories using the validated OST as a common reference tool. Not surprisingly there was a general understanding that severe cases of PSCs needed a more comprehensive treatment and health care utilization than mild cases of PSCs. Usually patients with mild PSCs only have slight skin changes, involving a small area of the skin and requiring no active treatment. Based on French unit costs treatment of a ‘mild’ PSC was estimated to add extra cost in the range of 19–40€ per patient for a seven week period. The main primary cost is for consultation fees. For moderate PSC, definite skin changes are seen, e.g. ulcers in the peristomal region and usually a larger area is involved. Furthermore non-prescription treatment may be required. The model based estimated extra cost for a moderate PSCs was in the range of 23–88€ per patient over ostomies without PSCs. For severe PSCs immediate attention is often needed e.g. systemic steroids or anti-bacterial treatment [18]. A severe condition often involves the whole skin surface beneath the appliance system and complicates the adhesion of the baseplate to the skin. Therefore a severe PSC has a significantly higher estimated added cost in the range of 106–196€ per patient for a seven week treatment period.

To determine the distribution of treatment cost for the different diagnostic categories of PSCs and the level of severity, the created model was applied on the real-life baseline data from the DS study as it is a large and well described published study – the largest of its kind including 3017 participants with a colostomy, ileostomy or urostomy [16]. The results showed that the overall average cost for an estimated seven week treatment period (incl. appliances and accessories) was 263€ per patient (n = 1742) for those with PSCs and 215€ per patient for those without PSCs. This is an additional cost of almost 50€ for those with PSCs for a seven week treatment period. France has approximately 114,000 people with ostomies (according to survey conducted by the French association of stoma care nurses (A.F.E.T.)). Given the prevalence of PSCs of 60% in the Dialogue Study requiring PSC treatment, this represents 3.4 million euros of additional costs over seven weeks and 25.4 million euros yearly in France, assuming that all patients with a stoma have had adequate access to stoma care nursing. Fifteen percent of the participants from the DS suffered from severe PSCs. The treatment cost for this group was estimated to be 6.1 fold higher compared with ‘mild’ cases and 4.5 fold higher compared with ‘moderate’ cases. It is also the case for pre-existing skin diseases including atopic dermatitis and psoriasis that often become aggravated on the peristomal skin and exacerbate the severity of the complication and hereby the need for active treatment. The estimated treatment costs for the underlying diseases in the DS showed that ‘Allergic dermatitis’, ‘disease related’ and ‘infection related’ had the highest costs (70.3€, 102.0€ and 60.4€ respectively). People with stomas suffering from pre-existing skin diseases may benefit from more regular visits to a SCN to avoid more severe PSCs, subsequently reducing costs.

Unfortunately for many patients, PSCs may result in a vicious circle where the skin problem causes failure of the adhesive, which in turn gives rise to leakage and again can lead to more recalcitrant skin problems. Leakage is particularly problematic for patients with an ileostomy, because the condition of their skin is likely to deteriorate rapidly following leakage [18]. The absence of colonic function in patients with an ileostomy leads to more frequent stool, thus resulting in a greater risk of skin irritation compared with individuals with a colostomy [19]. Unsurprisingly, peristomal leakage, PSCs and overall QoL are interconnected [6], [20] and the global results from the DS confirmed that leakage is a critical factor in the development of PSC [21]. Testing the influence of several co-variables on PSC costs, leakage turned out to have the highest impact in the present study. From a leakage perspective of ‘rarely/never’ to ‘always/often’ there was a statistical significant impact on PSC cost (p<0.00001) and from ‘sometimes’ to ‘rarely/never’, p = 0.0115.

The management of leakage, the involvement and support of a SCN and the use of an appropriate appliance and accessories could potentially save money over the long term. In addition the frequency and severity of PSCs has a major impact on a patient's quality of life and overall daily living. A key aspect in optimal stomal function relates to stomal construction. Two recent papers have reported on the importance of high quality stoma construction, including adequate stoma length to minimize leakage under the adhesive and consequently the possibility of PSC [3], [4].

The presented model gives an estimation of the cost associated with different forms of PSCs. A similar approach has not been published to date involving experts in the field from different professional perspectives. The sensitivity analysis of the model showed that the PSC cost estimate is robust to changes in all 15 diagnostic subcategories.

### Limitations of the study

The model outlined, and the estimated costs, however has a number of limitations. All treatment estimates are based on a global understanding from expert experiences applied to average PSC cases and not on real-life observations. If local treatment differs significantly from the global understanding it should be considered to adjust the treatment algorithm accordingly. The estimated costs are for a seven week treatment nevertheless the analysis does not address the outcome of the PSC after this period. In France, only four out of the 12 accessories recorded in the DS are paid by the health care system and therefore only these are incorporated in the total cost estimations. As all costs are based on French unit cost a re-estimation should be considered if local unit cost deviates significantly from French costs.

Several of these limitations could be addressed by a future real-life study following stoma patients and their PSCs over a longer period e.g. six months to explore the precise effect of treatment, extent of recurrent PSC and the appropriate resources needed.

PSCs are a common post-operative complication. It affects the patient physically and psychologically, ultimately prolonging rehabilitation and adaptation to the stoma. Furthermore PSCs significantly increase the cost both for society but also for the individual living with a stoma. Ideally PSCs should be prevented and awareness of the data presented here is important to disclose to surgeons creating a stoma and to other stoma care professionals (e.g dermatologists) as the quality of stoma construction and management may have a life-long direct impact for the patient's well being and major financial implications for the health care system.

### Conclusion

PSCs are common and both frequency and severity are under-recognized and under-reported. A key causative factor is undoubtedly peristomal leakage and individuals with an ileostomy are most at risk from a combination of the higher output and irritant nature of the effluent as compared with those people with a colostomy.

The consequences of PSCs are substantial, both from the patient and the health economy viewpoint. The extent of the problem warrants a major focus on methods to minimize the risk, detect PSCs at an early stage and institute optimal treatment to prevent the long-term, debilitating and expensive complications.

## Supporting Information

Table S1 Health care interventions for the 5 diagnostic categories seen in a 6–8 week perspective. * 2× SCN, 50% 2× specialist consultations, infliximab, 25% week opiod.
(DOCX)

Table S2 (A) French Unit Costs. ^1^ Nomenclature generale des actes professionnels infirmiers, Site de l'assurance maladie, www.ameli.fr. 2011. ^2^ Tarifs conventionnels médecins spécialistes. Site de l'assurance maladie, www.ameli.fr, 2011. ^3^ Arrêté du 1er mars 2011 fixant pour l'année 2011 les éléments tariffaires mentionnés aux I et IV de l'article L. 162-22-10 du code de la sécurité sociale. Secteur privé.(www.legifrance.gouv.fr) - Classification Commune des Actes Médicaux (C.C.A.M). www.ameli.fr. 2011. ^4^ Assistance Publique des Hôpitaux de Paris (APHP). Service Financiers, Paris 2008. ^5^ BCB drugs database. Groupe Cegedim, Boulogne-Billancourt.www.resip.fr, 2011. (B) French ostomy appliance and accessories cost. The cost varies with the type of product in accordance with the following: • reimbursement prices are regarded as independent of manufacturer. • individual components of the appliance are priced thus (arbitrary units): One piece convex appliances are not reimbursed in France. The relative difference between two piece flat and convex base plates has been added to the cost of a one piece appliance in the DialogueStudy dataset for one piece convex appliances. Refence: Liste des produits et prestations remboursables 2011 (www.ameli.fr).
(DOCX)
