# Supplementary material for: Peristomal Skin Complications Are Common, Expensive, and Difficult to Manage: A Population Based Cost Modeling Study
Source: PLoS One. 2012 May 24;7(5):e37813. doi: 10.1371/journal.pone.0037813 (PMC3359986; doi:10.1371/journal.pone.0037813)
Supplement: Table S1 — Health care interventions for the 5 diagnostic categories seen in a 6–8 week perspective. * 2× SCN, 50% 2× specialist consultations, infliximab, 25% week opiod. (DOCX) [file pone.0037813.s001.docx]

**Supporting information table S1**

|  | **%** | **Irritant contact dermatitis** |
| --- | --- | --- |
| **Mild** | 100 | SCN consultation |
|  | 40 | 2nd SCN consultation |
| **Moderate** | 100 | SCN consultation |
|  | 51 | 2nd SCN consultation |
|  | 15 | 3rd SCN consultation |
|  | 6 | Topical corticosteroid |
| **Severe** | 100 | SCN consultation |
|  | 98 | 2nd SCN consultation |
|  | 59 | 3rd SCN consultation |
|  | 58 | 4th SCN consultation |
|  | 6 | Topical corticosteroid |
|  | 3 | Local surgical revision (ambulatory)  Re-configuration of stoma height, removal of skin flaps, stoma stenosis |
|  | 2 | Redo-surgery (full surgery)  Stoma re-sitting, complete stoma repair |
|  | 11 | Additional home care |

|  | **%** | **Allergic dermatitis** |
| --- | --- | --- |
| **Mild** | 100 | SCN consultation |
|  | 100 | 2nd SCN consultation |
|  | 21 | Dermatologist consultation |
|  | 16 | 2nd dermatologist consultation |
|  | 7 | 3rd dermatologist consultation |
|  | 56 | Topical corticosteroid |
| **Moderate** | 100 | SCN consultation |
|  | 100 | 2nd SCN consultation |
|  | 14 | 3rd SCN consultation |
|  | 46 | Dermatologist consultation |
|  | 25 | 2nd dermatologist consultation |
|  | 16 | 3rd dermatologist consultation |
|  | 9 | 4th dermatologist consultation |
|  | 9 | Systemic prednisolone |
|  | 66 | Topical corticosteroid |
| **Severe** | 100 | SCN consultation |
|  | 100 | 2nd SCN consultation |
|  | 14 | 3rd SCN consultation |
|  | 7 | 4th SCN consultation |
|  | 68 | Dermatologist consultation |
|  | 47 | 2nd dermatologist consultation |
|  | 29 | 3rd dermatologist consultation |
|  | 27 | 4th dermatologist consultation |
|  | 20 | Systemic prednisolone |
|  | 41 | Topical corticosteroid |
|  | 6 | Additional home care |

|  | **%** | **Mechanical trauma** |
| --- | --- | --- |
| **Mild** | 100 | SCN consultation |
|  | 24 | 2nd SCN consultation |
|  | 1 | 3rd SCN consultation |
| **Moderate** | 100 | SCN consultation |
|  | 29 | 2nd SCN consultation |
|  | 20 | 3rd SCN consultation |
|  | 12 | Bandage to treat ulceration (wound dressing) |
| **Severe** | 100 | SCN consultation |
|  | 93 | 2nd SCN consultation |
|  | 27 | 3rd SCN consultation |
|  | 26 | Bandage to treat ulceration (wound dressing) |
|  | 1 | Local surgical revision (ambulatory)  Re-configuration of stoma height, removal of skin flaps, stoma stenosis |
|  | 1 | Redo-surgery (full surgery)  Stoma re-siting, complete stoma repair |
|  | 14 | Additional home care |

|  | **%** | **Disease related** |
| --- | --- | --- |
| **Mild** | 100 | SCN consultation |
|  | 96 | SCN conference with dermatologist or gastroenterologist. |
|  | 71 | 2nd SCN consultation |
|  | 26 | Topical corticosteroid |
| **Moderate** | 100 | SCN consultation |
|  | 100 | SCN conference with dermatologist or gastroenterologist. |
|  | 66 | 2nd SCN consultation |
|  | 59 | Specialist consultation (dermatologist, gastroenterologist, Gastro-Intestinal (GI) surgeon) |
|  | 24 | 2nd specialist consultation |
|  | 58 | Topical corticosteroid |
|  | 11 | Immunosuppressives (50% tacrolimus, 50% Cyclophosphamide) |
|  | 9 | Systemic prednisolone |
|  | 2 | Pyroderma gangrenosum* |
| **Severe** | 100 | SCN consultation |
|  | 100 | SCN conference with dermatologist or gastroenterologist. |
|  | 100 | 2nd SCN consultation |
|  | 96 | Specialist consultation (dermatologist, gastroenterologist, GI surgeon) |
|  | 67 | 2nd specialist consultation |
|  | 44 | 3rd specialist consultation |
|  | 86 | Topical corticosteroid |
|  | 22 | Immunosuppressives (50% tacrolimus, 50% Cyclophosphamide) |
|  | 24 | Systemic prednisolone |
|  | 3 | Pyroderma gangrenosum* |
|  | 7 | Additional home care |
|  | 5 | Hospitalisation |

* 2 x SCN, 50 % 2 x specialist consultations, infliximab, 25% week opiod.

|  | **%** | **Infection related** |
| --- | --- | --- |
| **Mild** | 100 | SCN consultation |
|  | 25 | 2nd SCN consultation |
|  | 27 | SCN conference w. specialist (derm., GI surgeon., gastroentero.) |
|  | 15 | Specialist consultation |
|  | 15 | 2nd specialist consultation |
| **Moderate** | 100 | SCN consultation |
|  | 65 | 2nd SCN consultation |
|  | 59 | SCN conference w. specialist (derm., GI surg., gastroentero.) |
|  | 24 | Specialist consultation |
|  | 12 | 2nd specialist consultation |
| **Severe** | 100 | SCN consultation |
|  | 100 | 2nd SCN consultation |
|  | 97 | SCN conference w. specialist (derm., GI surg., gastroentero.) |
|  | 100 | Specialist consultation |
|  | 97 | 2nd specialist consultation |
|  | 80 | Systemic anti fungal treatment |
|  | 20 | Systemic antibiotics |
|  | 5 | Hospitalisation |
|  | 8 | Redo-surgery (full surgery)  Stoma re-siting, complete stoma repair |
