# Supplementary material for: Peristomal Skin Complications Are Common, Expensive, and Difficult to Manage: A Population Based Cost Modeling Study
Source: PLoS One. 2012 May 24;7(5):e37813. doi: 10.1371/journal.pone.0037813 (PMC3359986; doi:10.1371/journal.pone.0037813)
Supplement: Table S2 — (A) French Unit Costs. 1 Nomenclature generale des actes professionnels infirmiers, Site de l'assurance maladie, www.ameli.fr. 2011. 2 Tarifs conventionnels médecins spécialistes. Site de l'assurance maladie, www.ameli.fr, 2011. 3 Arrêté du 1er mars 2011 fixant pour l'année 2011 les éléments tariffaires mentionnés aux I et IV de l'article L. 162-22-10 du code de la sécurité sociale. Secteur privé.(www.legifrance.gouv.fr) - Classification Commune des Actes Médicaux (C.C.A.M). www.ameli.fr. 2011. 4 Assistance Publique des Hôpitaux de Paris (APHP). Service Financiers, Paris 2008. 5 BCB drugs database. Groupe Cegedim, Boulogne-Billancourt.www.resip.fr, 2011. (B) French ostomy appliance and accessories cost. The cost varies with the type of product in accordance with the following: • reimbursement prices are regarded as independent of manufacturer. • individual components of the appliance are priced thus (arbitrary units): One piece convex appliances are not reimbursed in France. The relative difference between two piece flat and convex base plates has been added to the cost of a one piece appliance in the DialogueStudy dataset for one piece convex appliances. Refence: Liste des produits et prestations remboursables 2011 (www.ameli.fr). (DOCX) [file pone.0037813.s002.docx]

**Supporting information table S2**

(A)

| **Resources** | **Unit** | **Unit cost**  **€** | **Reference** |
| --- | --- | --- | --- |
| SCN (First visit) | Visit | 14.9 | ^1^ |
| SCN (General visit) | Visit | 14.9 | ^1^ |
| SCN/specialist conference | Session | 14.9 | ^1^ |
| Additional home care | Visit | 29.8 | ^1^ 7 visits |
| Dermatologist (First visit) | Visit | 44.0 | ^1^ |
| Dermatologist (General) | Visit | 28.0 | ^2^ |
| Gastro-surgeon (First visit) | Visit | 44.0 | ^2^ |
| Gastro-surgeon (General) | Visit | 28.0 | ^2^ |
| Gastroenterologist (First visit) | Visit | 44.0 | ^2^ |
| Gastroenterologist (General) | Visit | 28.0 | ^2^ |
| Local surgical revision | Case | 520 | ^3^ |
| Redo-surgery | Case | 4437 | ^3^ |
| Hospital stay | Day | 546 | ^4^ |
| Wound dressing | Once | 3.05 | ^5^ Algoplaque 10x10 cm |
| Topical corticosteroids | Regiment | 2.57 | ^5^ Hydracort 0.5% 30g |
| Prednisolone | Regiment | 4.89 | ^5^ Solupred 20 mg |
| Topical anti fungal drug | Regiment | 1.94 | ^5^ Fungizone lotion 30 ml |
| Systemic anti fungal drug | Regiment | 8.78 | ^5^ Amphotericine 250 mg |
| Systemic antibiotics | Regiment | 6.3 | ^5^ Amoxicilline 500 mg |
| Tacrolimus | Regiment | 34.02 | ^5^ Protopic pommade 30g 0.03% |
| Cyclophosphamide | Regiment | 12.74 | ^5^ Endoxan 50 mg |
| Infliximab | Regiment | 492.81 | ^5^ Remicade 100mg/20ml x 3 |
| Weak opiods, systemic | Week | 8.44 | ^5^ 2 weeks treatment |

(B)

| **Appliance/accessories** | | **Cost**  **€** | **Assumed usage** |
| --- | --- | --- | --- |
| 1 piece, closed bag | | 2.37 | 12 hours |
| 1 piece, open bag | 3.19 | | 24 hours |
| 2 piece, mechanical coupling, base plate | 3.48 | | 55 hours |
| 2 piece, mechanical coupling, convex base plate | 4.91 | | 55 hours |
| 2 piece, mechanical coupling, open bag | 2.27 | | 24 hours |
| 2 piece, mechanical coupling, closed bag | 1.44 | | 12 hours |
| 2 piece, adhesive coupling, base plate | 3.48 | | 55 hours |
| 2 piece, adhesive coupling, convex base plate | 4.91 | | 55 hours |
| 2 piece, adhesive coupling, open bag | 2.64 | | 12 hours |
| 2 piece, adhesive coupling, closed bag | 1.81 | | 24 hours |
| Paste or similar | 11.13 | | 60 gram for 3 weeks |
| Protective sheet dressing | 2.86 | | 10 sheets per month |
| Belt | 3.42 | | 6 months |
